# Evaluating True BCI Communication Rate through Mutual Information and Language Models

**DOI:** 10.1371/journal.pone.0078432

**Published:** 2013-10-22

**Authors:** William Speier, Corey Arnold, Nader Pouratian

**Affiliations:** 1 Department of Bioengineering, University of California Los Angeles, Los Angeles, California, United States of America; 2 Department of Neurosurgery, University of California Los Angeles, Los Angeles, California, United States of America; 3 Interdepartmental Program in Neuroscience, University of California Los Angeles, Los Angeles, California, United States of America; 4 Brain Research Institute, University of California Los Angeles, Los Angeles, California, United States of America; 5 Medical Imaging Informatics Group, University of California Los Angeles, Los Angeles, California, United States of America; The University of Plymouth, United Kingdom

## Abstract

Brain-computer interface (BCI) systems are a promising means for restoring communication to patients suffering from “locked-in” syndrome. Research to improve system performance primarily focuses on means to overcome the low signal to noise ratio of electroencephalogric (EEG) recordings. However, the literature and methods are difficult to compare due to the array of evaluation metrics and assumptions underlying them, including that: 1) all characters are equally probable, 2) character selection is memoryless, and 3) errors occur completely at random. The standardization of evaluation metrics that more accurately reflect the amount of information contained in BCI language output is critical to make progress. We present a mutual information-based metric that incorporates prior information and a model of systematic errors. The parameters of a system used in one study were re-optimized, showing that the metric used in optimization significantly affects the parameter values chosen and the resulting system performance. The results of 11 BCI communication studies were then evaluated using different metrics, including those previously used in BCI literature and the newly advocated metric. Six studies' results varied based on the metric used for evaluation and the proposed metric produced results that differed from those originally published in two of the studies. Standardizing metrics to accurately reflect the rate of information transmission is critical to properly evaluate and compare BCI communication systems and advance the field in an unbiased manner.

## Introduction

Brain computer interface (BCI) systems convert neurological signals into computer commands in order to restore function to patients who have lost control of effector muscles. Several BCI systems are currently under development, with applications that include moving a cursor on a screen, controlling a robotic prosthesis, and typing letters and words to restore communication [1]. With several groups working diligently to advance these technologies, regardless of application, it is paramount to have validated metrics with appropriate assumptions to compare between system designs and move the field forward in an unbiased manner. In this work, we focus on BCI for restoring language communication and the associated metrics for evaluation.

The P300 speller is the most commonly used BCI approach for restoring linguistic communication [2]. Briefly, a user observes a grid of characters on a computer screen (analogous to a visual keyboard) while subsets of characters are flashed in pseudo-random patterns. These flashes result in visual stimuli that elicit evoked electroencephalographic (EEG) responses which are then used to decipher the target letter or symbol of interest. System noise requires that multiple stimulus presentations be averaged in order to achieve sufficient signal-to-noise to make accurate selections, resulting in slow typing rates. Several approaches have been developed to improve performance, including using different stimulus paradigms [3]–[5], optimizing system parameters [6]–[8], implementing different classifiers [9]–[11], and integrating language domain knowledge [5], [12], [13]. Alternative methodologies to the P300 speller have also been explored, including auditory stimuli [14], [15], and different neurological phenomena such as motor imagery [16] and steady state visually evoked potentials (SSVEP) [17], [18].

Given the number and variety of approaches, a reliable metric is important for evaluation and comparison across experimental paradigms and ultimately across studies, which to date is lacking. A useful metric must consider the amount of time taken, the accuracy of selections, and the tradeoff between the two. Increasing the amount of data and therefore time needed to make a decision can increase the accuracy of the selection at the expense of system speed. Perfect accuracy however is not always necessary as a BCI can integrate prior knowledge about the domain and common user behavior to understand output despite errors. In the case of typing natural language, for instance, text is often readable despite the presence of typos. In a non-typing context, errors may not be permissible, so errors must be corrected by subsequent “undo” selections, which would result in a perfect accuracy, but slower typing speed.

Information Transfer Rate (ITR) is a general evaluation metric devised for BCI systems that determines the amount of information that is conveyed by a system's output [19]. The metric is appealing for several reasons: it is derived from information theory principles, it combines the competing statistics of speed and accuracy, and it reduces to an information transfer problem that can be compared across applications [20]. However, ITR is not appropriate for evaluating language systems because it makes two assumptions that are incorrect in general, particularly in the language domain: 1) that all possible selections are equally probable and 2) that systems are memoryless. Several methods have since been introduced in attempt to reduce the adverse attributes of ITR. Word Symbol Rate (WSR) normalizes ITR by its maximum value and then scales down based on error rate [14]. Practical Bit Rate (PBR) finds the theoretical bit rate if the user had corrected every selection error [3]. Characters per Minute (CPM) calculates the theoretical number of characters correctly typed after error correction [7]. Output Characters per Minute (OCM) is an online metric similar to CPM that requires all errors to be corrected [12]. In general, these metrics all depend on aspects that are system specific and therefore not generalizable (see methods).

A standard method for evaluating results does not exist, making it difficult to compare the relative value or the superiority of different experimental paradigms or approaches. We present an information rate metric (MI_n_) based on mutual information designed to incorporate language domain knowledge to more accurately measure the utility of language-based BCI systems. Three versions of this metric are compared to five existing methods that are currently used for evaluation in P300 literature. We use each metric to optimize the dataset used by Speier et al. [13] to show the difference in performance achieved. We then reevaluate the results of 11 published studies using the existing metrics used in the literature and compare the results to those determined using the proposed metrics. We cannot retroactively account for differences in system parameters and experimental paradigms, so it is impossible to make fair comparisons between studies. However, we show the effects of choosing various evaluation metrics on comparisons made within studies and the conclusions that result. Our analysis shows that the selection of a metric significantly affects system optimization as well as the evaluation of different approaches for BCI communication, leading to the necessity for adopting a consistent and reliable performance metric.

## Methods

Data from published BCI communication studies are used to show the effects of evaluation (Table 1). Studies were included if they provided the accuracy and selection speed that were achieved by each study subject, which are the only two values necessary for calculating each evaluation metric, allowing us to evaluate each subject's performance separately using each metric. The average values were then taken for each study arm and the results were reanalyzed. The results of each of these studies were evaluated using both previously published as well as the proposed metrics.

**Table 1:** Parameter values, optimization metric, and evaluation metric used in each of the included datasets.

Study	Method	Subjects	N	Steps	ISI (ms)	Gap (s)	Opt	Eval
Kaper (22)	all	8	36	1	140	2	ITR	ITR
Serby (11)	all	6	36	1	150	2	ITR	SR, ACC, ITR
Blankertz (16)		2	6	2	NA	NA	none	OCM
Sellers (6)	3×3,175	5	9	1	175	5	none	ITR
	3×3,350	5	9	1	350	5	none	ITR
	6×6,175	5	36	1	175	5	none	ITR
	6×6,350	5	36	1	350	5	none	ITR
Furdea (14)	auditory	13	25	1	625	3.75	WSR	ITR, WSR
	visual	13	25	1	287.5	8.75	WSR	ITR, WSR
Ceocetti (17)		8	5	≥3	NA	NA	none	ACC, ITR, OCM
Townsend (3)	all	18	72	1	125	3.5	WSR	ITR, PBR
Jin (4)	all	10	84	1	175	2	none	ACC, PBR
Ryan (12)	all	24	72	1	125	6	WSR	ITR, OCM
Schreuder (15)	S1	14	6	2	175	18.25	none	ACC, ITR, OCM
	S2a, S2b	14	6	2	175	12	none	ACC, ITR, OCM
Speier (13)	all	6	36	1	125	3.5	ITR	ITR

### Evaluation with Previously Published Metrics

Means for evaluating published studies using previously described metrics are briefly described here. Please see SI1-SI5 for derivations.

#### Information Transfer Rate

ITR finds the average bits of information contained in each selection, 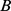, as the mutual information between the selection, 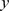, and the target character, 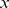, divided by the time required. The method assumes that each selection is independent, marginal probabilities are uniform over the character in the grid (i.e., 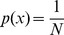 where 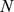
is the number of possible selections), and errors are uniform over the non-target characters (i.e., 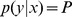 if 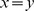 and
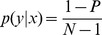
 otherwise where 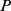 is the selection accuracy).
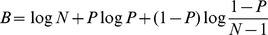



ITR is then the bits per symbol divided by the average time required to select a single symbol, 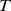.
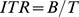



**Figure 1:**
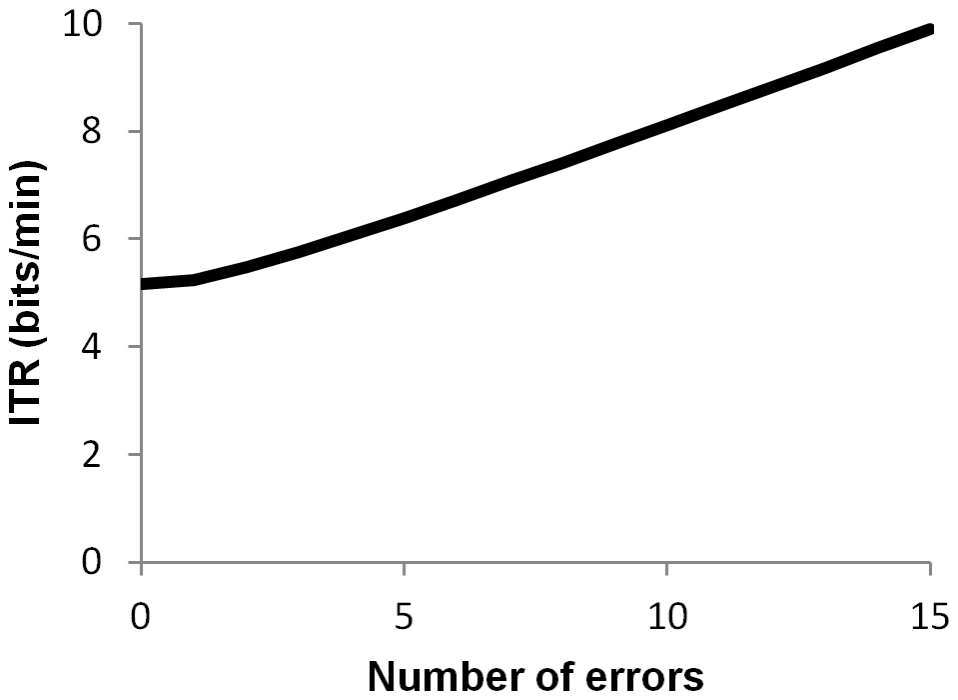
ITR calculation for hypothetical cases of typing a 10 character sequence with error correction in 10 minutes. For each error, two additional selections are required. As a result, the ITR increases because the increase in number of selections more than offsets the decrease in selection accuracy.

The theory behind ITR was derived from the concept of a noisy channel with 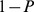 representing the error frequency in the output string. Instead, BCI literature generally uses 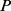 as the selection accuracy. In some systems, this is equivalent, but it is not in cases where multiple steps are used for one selection or where backspaces can be used to correct errors. In these cases, counterintuitive results can occur where two users can type the same string without errors and one can have a slower speed, but a higher ITR (Figure 1).

#### Word Symbol Rate

To calculate WSR, the bits per symbol are scaled by their maximum possible value, 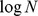. The result is called symbol rate (SR), which is treated as the probability of a correct selection, which is not appropriate when multiple decisions are required for a selection. The average number of selections necessary to choose one character is then found by determining the number of additional selections required for correcting errors. If an average selection provides less than half the maximal amount of information, then there will be more errors than correct selections, so WSR becomes,
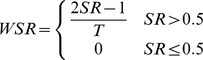



#### Practical Bit Rate

PBR also simulates the correction of all typing errors. However, instead of using SR, actual typing accuracy is used. This metric then divides the bits of information contained in a single correct selection (still assuming all characters have equal probability) by the average number of selections required to choose that character. Subjects with selection accuracy below 50% would make errors at a faster rate than they would be able to correct them, so the bit rate becomes,
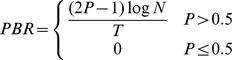



Practical bit rate has also been computed substituting ITR for 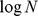 [4]. Because both PBR and ITR include penalties for incorrect selections, this metric will double count errors, resulting in an overly conservative estimate of bit rate.

#### Characters per Minute

CPM extends PBR as it uses the same correction factor to account for additional selections required to correct errors. It differs in that it does not take matrix size into account and instead calculates the number of characters selected per minute.
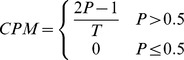



#### Output Characters per Minute

OCM is only possible in online implementations, requiring all errors to be corrected. OCM is computed by dividing the total number of typed characters by the time required to type them.

### Proposed Method

We propose an alternate mutual information-based metric that has similar benefits to ITR, but does not rely on the same assumptions. Three versions are included that progressively remove assumptions, resulting in increasingly accurate representations of the true bit rate. The first version, MI_0_, removes the uniform probability assumption and instead uses relative symbol frequency as a prior probability. The second version, MI_n_, removes the assumption of independent selections by incorporating knowledge about the 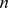 previous characters using an n-gram model. The third version, MI_ne_, uses an error model to incorporate additional information contained in incorrect selections (see SI6-SI8 for derivations).

This metric is applicable in any case where the selection probabilities can be modeled. In general, this can be done by measuring relative frequencies of different selections. In many contexts, this data is not widely available, but it can be learned by measuring the selection frequencies as a user interacts with the system. In the context of natural language, these probabilities can easily be estimated by measuring relative character frequencies in a corpus of natural language. For this reason, and because these systems are traditionally evaluated in pure spelling mode, evaluating is performed here in a language context. This method could easily be extended to any system with an available data set of past output.

#### MI_0_

**Figure 2:**
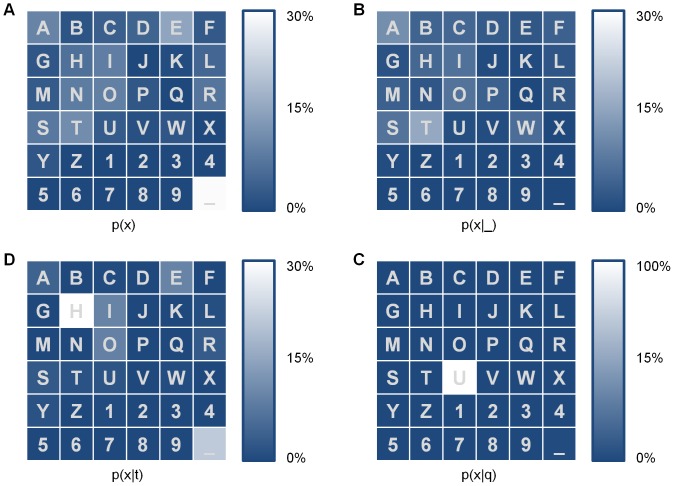
Marginal probability of characters in the English language (a) and conditional probabilities of characters given previous characters of space (b), ‘t’ (c), and ‘q’ (d).

With this method, the system remains memoryless (i.e., all selections are assumed independent) and all errors are still assumed to be uniform over all incorrect characters. Similar to ITR, MI_0_ is the mutual information between the target symbol and the selected symbol. However, we remove the assumption that all characters are equally probable and instead determine their probabilities by their relative frequencies in the general purpose Brown corpus [21] (Figure 2) as
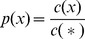
where 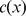 is the number of occurrences of character 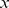 in the corpus and 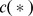 is the total number of characters in the corpus. The bits per symbol may then be computed:
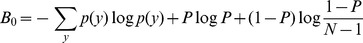
where 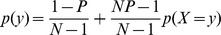. Note that here 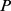 represents the accuracy in the final output, not the individual selection accuracy. Multiplying by the size of the output string and dividing by the total time yields MI_0_:
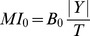



#### MI_n_

MI_n_ builds on MI_0_ by removing the assumption that all character selections are independent. We assume that selected characters are directly dependent on the respective target characters and that target characters are dependent on the previous n characters. The conditional probabilities 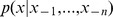 can be found by determining the fraction of occurrences of the string 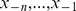 that are followed by 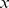 in the corpus:
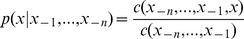



Knowledge from additional steps can be factored into this equation by conditioning over previous targets and summing over their possible values as follows:
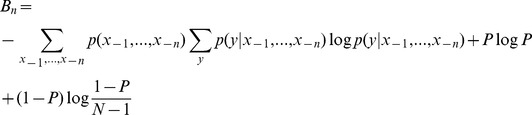
where 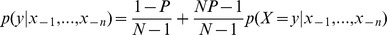. Multiplying by the size of the output string and dividing by time yields the value for MI_n_.
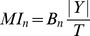



#### MI_ne_

Townsend et al. showed that errors in P300 systems are systematic, and therefore incorrect selections contain information about the target character [3]. Below, we propose error models based on values determined in their analysis. First, errors in the checkerboard paradigm have been shown to occur more often within the same virtual matrix as the target character.
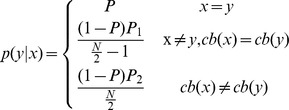



Where 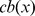 refers to the virtual matrix that character 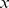 is assigned to, 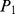 refers to the probability of an error occurring in the same virtual matrix as the target, and 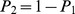 refers to the probability of an error occurring in a different virtual matrix. These values were found to be .7414 and .2586 respectively by Townsend et al. [3].

Next, there were three distinct types of errors found in the row/column paradigm. Adjacent characters were observed to be selected the most often, followed by characters that shared a row or column with the target character, both of which were more likely than erroneously selecting a distant character.
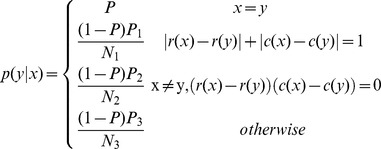



Here, 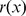 and 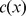 are the row and column of character 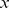 in the matrix. 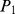, 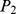, and 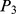 are the probabilities of incorrectly picking characters that are adjacent, in the same row or column, or anywhere else relative to the target character. 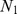, 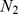, and 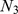 are the numbers of characters that are adjacent, in the same row or column, or anywhere else relative to the target character. The error probabilities were found to be .4065, .4452, and .1483 respectively by Townsend et al. [3].

Other flashing paradigms such as those presented by Jin et al. [4] are more random so error patterns are less likely to occur. No other papers included error analysis, so a uniform model was used for P300 systems with alternative flashing paradigms. The bits per symbol is then
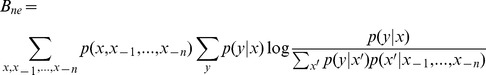



The information rate is then found by Multiplying by the size of the output string and dividing the bits per symbol by the total time.
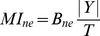



### Analysis

#### Data

Eleven studies were chosen as a representative sample of existing BCI communication literature. Seven visual P300 studies were included: one study focused on optimizing system parameters [6], two proposed new flashing paradigms [3], [4], two used novel classification techniques [22], [11], and two integrated language information [12], [13]. The remaining four studies proposed systems based on alternative neurological signal paradigms including audio P300 [14], [15], motor imagery [16], and SSVEP [17]. Nine of the studies [3]–[4], [11]–[15], [22] included comparisons between study arms to validate the proposed method. The remaining two [16], [17] each demonstrated their system alone as a proof of concept.

The studies reviewed used a variety of system parameters (Table 1), all of which significantly influence system performance. Because these values vary widely, performance differences observed in a comparison across studies could be a result of the different parameter combinations, rather than a validation of the techniques used. Additionally, each study used a different subject population and sample sizes were small (between two and 24), making it difficult to find significant differences in results. Individual studies are usually self-controlled and use standardized systems, which alleviates these concerns. We therefore focus on reanalyzing the comparisons within studies instead of comparing results between studies. Comparison across studies becomes more appropriate in situations where a study builds directly upon a previous one, which allowing limiting the parameter and protocol variation.

Each aforementioned study was evaluated using the each of the existing and proposed evaluation metrics. Within each study, the results of the different groups were compared using paired t-tests. These results were then compared to the findings in the original paper.

#### Optimization

The first analysis performed considered a previously published dataset [13]. In this study, the probability of each of the possible characters was computed after each stimulus and the most probable character was selected once a confidence threshold was reached. In the published results, the value for the threshold was determined by choosing the value that optimized the results using the ITR metric.

Analysis consisted of re-optimizing the results using each of the metrics detailed above. The new optimal threshold probability is reported for each optimization as well as the corresponding performance using the MI_2e_ metric. These values are then compared to the results from optimizing on the MI_2e_ metric and evaluated for significance using paired t-tests.

## Results

### Optimization

**Figure 3:**
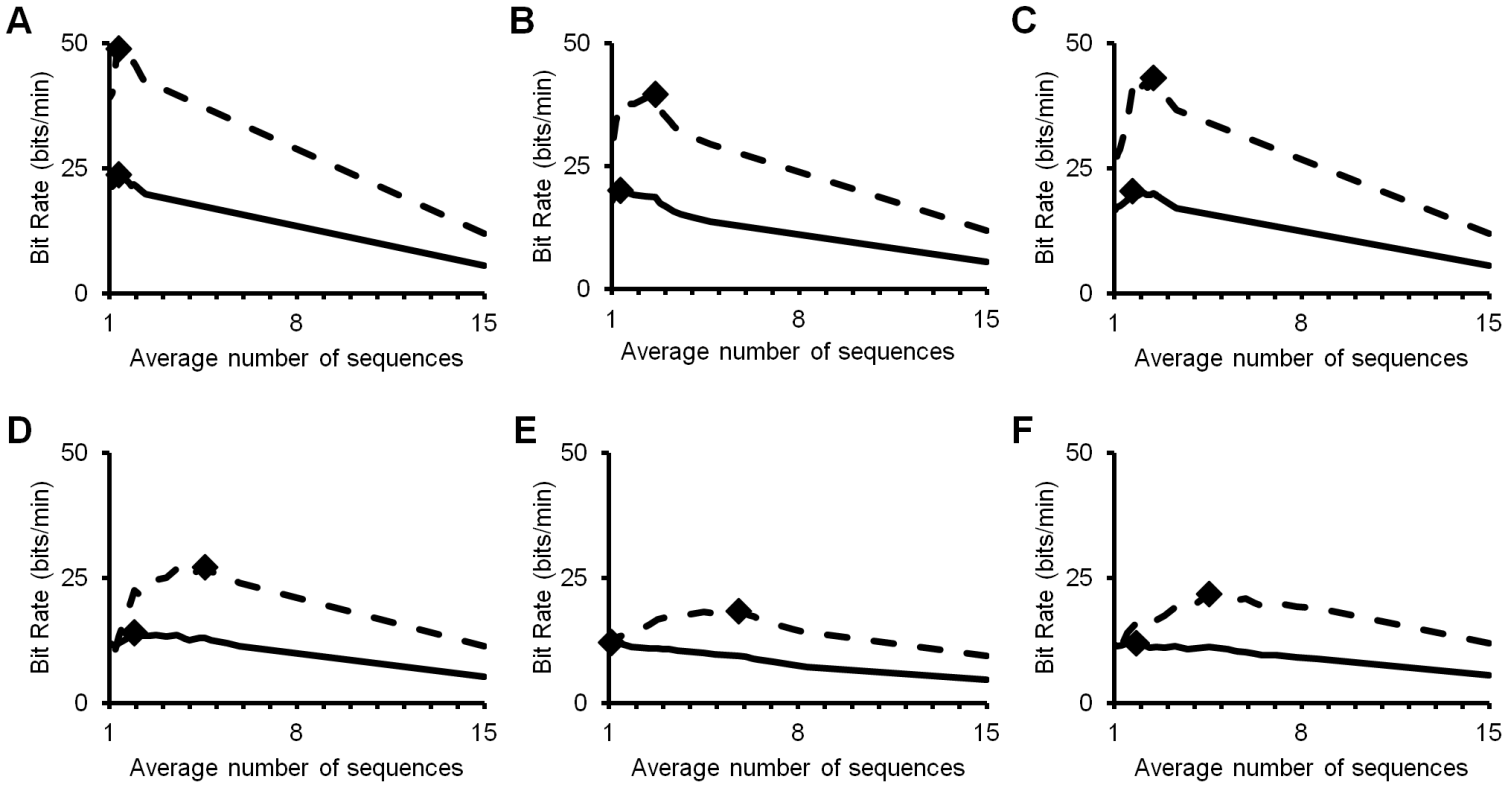
Values of ITR (broken curve), and MI_2e_ (full curve) versus the number of stimulus sequences used in classification for each subject in the Speier et al. (13) dataset (a–f). Optimal values are marked by diamonds.

The original optimization reported in Speier et al. used ITR and chose an optimal value of 0.86 for the threshold probability [13]. Many of the existing metrics chose similar optimal values, with only studies optimizing based on sample rate and accuracy choosing significantly different values. Using MI_0_ resulted in the same optimal values, and MI_2_ resulted in values that were lower, but not significant (p = 0.087) (Table 2). The threshold values chosen using MI_2e_ were significantly lower than those using all other metrics, with lower values for five of the six subjects (Figure 3).

**Table 2:** Threshold values and average MI_2e_ value of the dataset from Speier et al. when optimizing on different evaluation metrics.

Metric	Threshold	p-value	Bit rate	p-value
MI_2e_	0.49		17.05	
MI_2_	0.82	0.005	16.24	0.021
MI_0_	0.86	0.006	16.05	0.020
ACC	0.98	0.001	13.14	0.007
SR	0.12	0.003	15.25	0.008
ITR	0.86	0.006	16.05	0.020
WSR	0.93	0.001	15.31	0.005
PBR	0.87	0.005	15.97	0.015
CPM	0.87	0.005	15.97	0.015
OCM	*	*	*	*

OCM was not computable because the system did not require all errors to be corrected. p-values are determined using paired t-tests between the given value and the results when optimizing on MI_2e_. Asterisks denote methods that cannot be computed for the target system.

* method cannot be computed for the target system

When optimizing on the existing metrics, the average confidence threshold values varied between 0.12 and 0.98, and the corresponding information rates varied between 13.14 and 16.05 bits per minute. The optimized values achieved using MI_0_ were identical to those using ITR, and MI_2_ achieved an insignificant increase in results (p = 0.087). When optimizing on MI_2e_, the average confidence threshold was significantly lower (0.49) and the derived bit rate (17.05) was significantly higher than those using any other metric (Table 2).

### Evaluation

In the Kaper et al. [22] study, all metrics other than WSR reflect better results using inner cross validation with significant differences between “inner” and “outer” noted using ITR, MI_0_, MI_2_, and MI_2e_ (p = 0.00044, p = 0.00033, p = 0.00027, and p = 0.00014, respectively), which is consistent with the published conclusions (Table 3).

**Table 3:** Results from published P300 papers reevaluated using different metrics.

Study	Method	ACC	SR	ITR	WSR	PBR	CPM	OCM	MI_0_	MI_2_	MI_2e_
Kaper (22)	**inner**	**54.38**	**13.85**	**25.21**	0.13	**9.41**	**1.82**	*	**21.13**	**12.94**	**18.06**
	outer	47.88	9.70	14.47	**0.23**	3.10	0.60	*	12.21	7.51	11.59
Serby (11)	ML	90.02	3.66	15.79	2.45	15.54	3.01	*	12.63	7.49	7.77
	**ICA**	**92.12**	**4.56**	**19.88**	**3.13**	**19.66**	**3.80**	*	**15.90**	**9.43**	**9.74**
	online	79.53	3.89	13.77	1.72	12.35	2.39	*	11.11	6.64	7.27
Blankertz (16)		*	*	*	*	*	*	4.88	19.95	11.73	11.73
Sellers (6)	**3×3,175**	61.25	**3.87**	**4.53**	**0.32**	**3.99**	**1.26**	*	2.43	1.58	1.82
	3×3,350	**69.38**	2.31	3.19	0.10	2.83	0.89	*	1.70	1.11	1.21
	6×6,175	53.75	2.31	4.50	0.26	2.68	0.52	*	**3.72**	**2.26**	**3.10**
	6×6,350	48.13	1.28	1.93	0.00	0.08	0.02	*	1.64	1.01	1.54
Furdea (14)	auditory	88.08	1.15	4.66	0.91	4.65	1.00	*	3.59	2.22	2.26
	**visual**	**98.08**	**3.54**	**15.75**	**3.24**	**15.79**	**3.40**	*	**12.15**	**7.46**	**7.49**
Ceocetti (17)		92.25	19.64	35.34	*	*	*	5.51	22.54	13.25	13.25
Townsend (3)	**cb72**	**91.52**	4.33	**23.01**	**3.12**	**22.45**	**3.64**	*	**15.67**	**9.25**	**9.28**
	rc72	77.34	**4.64**	19.70	2.07	16.51	2.68	*	13.68	8.12	8.59
Jin (4)	9-P	87.33	**5.82**	**29.32**	3.35	27.14	4.25	*	**18.65**	**11.09**	**11.09**
	12-P	88.00	5.40	27.49	3.20	25.97	4.06	*	17.48	10.39	10.39
	14-P	93.26	3.77	20.93	2.78	20.55	3.21	*	13.34	7.90	7.90
	**16-P**	93.23	5.26	29.14	**3.85**	**28.36**	**4.44**	*	18.58	11.00	11.00
	19-P	**93.99**	4.70	26.39	3.56	25.86	4.05	*	16.83	9.96	9.96
Ryan (12)	**PS**	84.92	**3.78**	17.85	2.03	16.46	2.67	**5.28**	**21.64**	**12.71**	**12.71**
	NS	**89.80**	3.74	**19.28**	**2.51**	**18.52**	**3.00**	3.12	12.79	7.51	7.51
Schreuder (15)	S1	**87.99**	2.08	3.75	*	*	*	0.62	2.54	1.49	1.49
	**S2a**	86.16	**3.05**	**5.27**	*	*	*	0.91	3.71	2.18	2.18
	**S2b**	86.07	2.96	5.26	*	*	*	**0.94**	**3.83**	**2.25**	**2.25**
Speier (13)	Static	82.97	5.91	22.06	2.65	20.24	3.91	*	17.78	10.60	11.42
	Dynamic	89.63	6.45	27.38	4.14	26.61	5.15	*	21.92	13.00	13.55
	**NLP**	**92.59**	**7.31**	**33.15**	**5.51**	**32.91**	**6.37**	*	**26.44**	**15.65**	**16.05**

Bold numbers refer to the leading method using that metric and bold method names refer to the results found in the original publication. Asterisks denote methods that cannot be computed for the target system.

* method cannot be computed for the target system

In the Serby et al. [11] report, all metrics agreed with the original conclusion that independent component analysis achieved a higher bit rate than the maximum likelihood method. Each metric showed significant results other than accuracy (p = 0.34) and WSR (p = 0.09) with p values ranging from 0.023 (PBR) to 0.008 (MI_2e_).

The Sellers et al. [6] paper showed varying results depending on the metric used. All existing metrics other than accuracy determined the 3×3 grid with an ISI of 175 ms to have the best performance, although none were significant. The three proposed metrics however identified the 6×6 grid with an ISI of 175 ms as the superior configuration with significant results (p = 0.015, p = 0.044, and p = 0.002, respectively).

All metrics in the Furdea et al. [14] study determined that the visual P300 speller was superior to their audio version. All metrics other than accuracy (p = 0.078) revealed significant differences between the two approaches with p values less than 10^−6^.

In the Townsend et al. [3] study, the results from the checkerboard paradigm proved better than the row/column paradigm on a 9×8 grid by all metrics other than selection rate. The results were significant using ITR (p = 0.035), MI_0_ (p = 0.044), and MI_2_ (p = 0.047), but not MI_2e_ (p = 0.12).

There was variability in the results of the system presented in Jin et al. [4]. The original paper concluded that 19-P method was superior using PBR, which is consistent with the WSR and CPM metrics. However, selection rate, ITR, MI_0_, MI_2_, and MI_2e_ all indicated the 9-P method was superior. In all cases, the results were close and none were statistically significant.

In the Ryan et al. [12] paper, evaluation using accuracy, ITR, SWR, PBR, or CPM revealed significantly higher values using the nonpredictive speller with p values between 0.02 and 0.04. OCM (the metric used in the original paper), MI_0_, MI_2_, and MI_2e_ all showed significantly higher rates for the predictive speller (p<10^−8^).

In the Schreuder et al. [15] paper, all metrics other than accuracy showed significantly higher results for the S2a and S2b trials, including ITR (the metric used in the original paper) and the proposed metric (p<0.0001). There were no significant differences between the S2a and S2b trials using any metric.

The Speier et al. [13] paper showed superior results for the NLP method regardless of the evaluation metric used. All metrics showed significant results other than selection rate (p = 0.064).

## Discussion

### Evaluation

In six of the 11 studies analyzed, changing the evaluation metric could have resulted in different conclusions from that originally published. Only two of the existing metrics, PBR and CPM, always agreed. This highlights the critical importance of identifying an appropriate metric for evaluation of P300 speller studies, and more generally all BCI studies.

The proposed metrics agreed with the published conclusion in nine of the 11 studies. In the Sellers et al. study, all existing metrics chose the 3×3 grid because they did not consider actual typing ability. Because they only have nine characters to choose from, their system would not be able to type most English words and is therefore less effective at communicating language. This shortcoming could be addressed by making selections in two steps, but the effectiveness would need to be reevaluated [6]. The proposed metrics also would have provided different conclusions in the study by Jin et al., although the results were close and the difference was not statistically significant [4].

Most existing metrics could not account for the predictive model used in the Ryan et al. [12] study. The nonpredictive speller achieved a higher accuracy and similar selection speed, so it was found to be significantly better in most cases. The only existing metric that was able to account for the improvements in their system was the metric introduced in the same paper. The metrics proposed here are able to account for the predictive model and thus agree with the highly significant results found in the study.

Another critical advantage of the proposed metrics is their universal utility. Only the proposed metrics were consistently computable and intuitive across all studies. Some of the existing metrics could not be computed for all of the studies either because all errors were not corrected (OCM), the system involved multi-step decisions (WSR, PBR, and CPM), or rates and accuracies were not recorded for intermediate steps (ITR). While ITR could be computed if all intermediate results were recorded, it did not always reflect actual performance. In the Schreuder et al. [15] study, subjects were able to type the target sentences faster in the S2b trial, but the S2a trial had a higher ITR value due to the multi-step nature of the system.

### Optimization

The performance of BCI systems is influenced highly by system parameter values. These parameters are typically set by optimizing using some metric. Our analyses illustrate the impact of the optimization method on system performance. Optimization is designed to make a value achieve its optimal value, so it is trivial that optimizing on MI_2e_ achieves the highest information rate. The interesting aspect of this analysis is the disparity between the threshold value determined by MI_2e_ and the thresholds chosen using other metrics. The threshold is significantly lower than the values determined by other metrics. A lower threshold results in faster decisions, resulting in significantly higher bit rates when error information is taken into account.

Optimizing on MI_2e_ results in the adoption of lower threshold values in part because it takes the information contained in errors into account. This information may not be useful in all cases. If the reader is not aware of the error model, then this information would be ignored and the functional information transfer would be that described by MI_2_. In this case, the optimization on MI_2e_ would be too aggressive, resulting in an error rate that might be too high for practical use. The end application should be considered when choosing the evaluation metric so that the system can be appropriately optimized.

In many BCI communication studies, optimization and evaluation are performed using different metrics. The papers referenced in this study used several different optimization procedures, resulting in incompatible results even after converting them to consistent metrics. Even within papers, various metrics are used for evaluation in order to compare with various different studies. Going forward, we suggest a standard metric should be chosen to standardize BCI results and allow for more consistent comparisons across studies, such as the one presented here.

### Error Model

The improvements in results from including the error model varied from negligible amounts [14] to over 50% improvement [6], and were based mainly on the average accuracy achieved. Depending on the application, information considered by this error model might not actually be useful. If the output string is sent to a post-processing algorithm designed to correct errors using this error model, it could be translated into a real increase in overall accuracy. When a human is reading the user input, knowledge of the trends of errors could be useful in trying to determine the attempted output, but this could be a difficult task. Further studies could show a reader's ability to correct different types of errors (see future directions). It is the system designer's role to consider the end application when determining the correct metric to use, and it might be appropriate to omit an error model in some instances.

### Limitations

The ideal error model used in MI_ne_ would include the actual probabilities 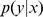 for all 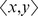 pairs for each subject. However, it would be impractical to actually find all of these in a training step, so some simplifying assumptions need to be made. The probabilities of adjacent, same row or column, and same virtual matrix probabilities used in the 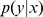 values used in section 2.2.3 would vary between subject and system, and therefore should be calculated during training rather than blindly using the values provided by Townsend et al. [3]. Unfortunately, studies rarely publish these numbers, so this was not possible in this study.

While adopting a standard evaluation metric makes information rates of BCI systems comparable, comparisons between studies can still be misleading. BCI systems are high-dimensional systems that can have many different parameters, electrode configurations, and hardware constraints. It is therefore difficult to determine whether an improved performance corresponds to a superior method or a better tuning of the system parameters. For this reason, researchers should be cautious when comparing between studies and limit these comparisons to situations where studies share similar configurations such as when a new study directly builds upon a previous one. Some work has been performed in parameter optimization [6]–[8], but several aspects such as the length of the pause between selections have not been addressed. Furthermore, most of these studies involved healthy subjects, so the translation of these results into the target patient population could vary between systems, irrespective of the evaluation metric used.

### Future Directions

In this study, we focused on using BCI systems for communicating language information. In general, these systems are often extended to include various types of menu-based commands [3]. Probabilities for selections can still be computed similar to the n-gram language model, assuming a data set of sequences of selections is provided. In this case, the conditional probability of a selection sequence would be the relative frequency of that sequence in the selection history. To our knowledge, no such data set has been published. Furthermore, all studies that we know of were performed using a pure spelling task. Studies of alternate uses of these systems would allow us to create more general models of selection probabilities in order to further generalize this metric.

To date, no BCI communication systems use information about the types of errors to improve their selections. Current systems treat all errors as a wrong answer that is either ignored or deleted, rather than combining it with knowledge about common types of errors to acquire information about the target symbol. Applications can improve their output if they incorporate this information through either a post-processing program or integrate it into the classifier itself. When designing a BCI system, constraints of the target domain should be considered because they provide information that can improve overall performance when incorporated into the classifier. To this end, we have recently reported the benefits of integrating knowledge of language into P300 speller classification [13].

Ultimately, the goal of a communication system is to convey the intent of the user. It is clear that a lower error rate is preferable, but it is uncertain how low it needs to be in order for the output to be understood. In addition to the number of errors, the types of errors that occur can be important to reader comprehension. In English, for instance, replacing a vowel with another vowel will often result in another word, while replacing a vowel with a consonant will usually result in a string that is not a word, making the error more apparent and easier to correct. The relationship between language-based BCI output accuracy and reader understanding has not yet been studied.

## Conclusion

The performance metric used is integral to the evaluation of BCI systems as it can influence optimization and comparison of different methods. Current methods for evaluating language-based BCI systems are largely misapplied and based on incorrect assumptions, leading to suboptimal implementations and misleading results. System designers should consider the inherent structure of the language domain and the ultimate goal of communication when developing and evaluating these systems. The mutual information metric presented here compensates for many of these shortcomings and provides a better way to compare and evaluate language-based BCI results.
